# The Intrinsic Biological Identities of Iron Oxide Nanoparticles and Their Coatings: Unexplored Territory for Combinatorial Therapies

**DOI:** 10.3390/nano10050837

**Published:** 2020-04-27

**Authors:** Vladimir Mulens-Arias, José Manuel Rojas, Domingo F. Barber

**Affiliations:** 1 Department of Immunology and Oncology, and NanoBiomedicine Initiative, Centro Nacional de Biotecnología (CNB)-CSIC, Darwin 3, Cantoblanco, 28049 Madrid, Spain; vmulens@cnb.csic.es; 2 Animal Health Research Center (CISA-INIA), Instituto Nacional de Investigación y Tecnología Agraria y Alimentaria, Valdeolmos, 28049 Madrid, Spain; rojas.jose@inia.es

**Keywords:** iron oxide nanoparticles, nanoparticle coatings, nanoparticle–macrophage interaction, nanoparticle–tumor cell interaction, nanoparticle–endothelial cell interaction

## Abstract

Over the last 20 years, iron oxide nanoparticles (IONPs) have been the subject of increasing investigation due to their potential use as theranostic agents. Their unique physical properties (physical identity), ample possibilities for surface modifications (synthetic identity), and the complex dynamics of their interaction with biological systems (biological identity) make IONPs a unique and fruitful resource for developing magnetic field-based therapeutic and diagnostic approaches to the treatment of diseases such as cancer. Like all nanomaterials, IONPs also interact with different cell types in vivo, a characteristic that ultimately determines their activity over the short and long term. Cells of the mononuclear phagocytic system (macrophages), dendritic cells (DCs), and endothelial cells (ECs) are engaged in the bulk of IONP encounters in the organism, and also determine IONP biodistribution. Therefore, the biological effects that IONPs trigger in these cells (biological identity) are of utmost importance to better understand and refine the efficacy of IONP-based theranostics. In the present review, which is focused on anti-cancer therapy, we discuss recent findings on the biological identities of IONPs, particularly as concerns their interactions with myeloid, endothelial, and tumor cells. Furthermore, we thoroughly discuss current understandings of the basic molecular mechanisms and complex interactions that govern IONP biological identity, and how these traits could be used as a stepping stone for future research.

## 1. Introduction

**Figure 1:**
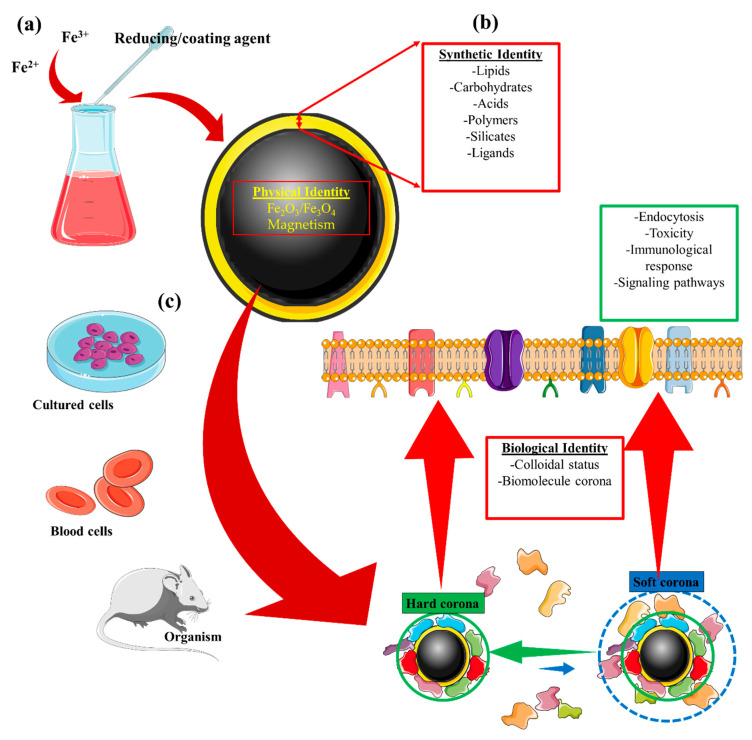
Iron oxide nanoparticle (IONP) identities and their relationships. (**a**) The core synthesis of IONPs provides the nanomaterial with its physical identity. For instance, the production of a small IONP (<20 nm) results in superparamagnetic nanoparticles. (**b**) The synthetic identity provides an added layer aimed at improving the functionality of the synthesized nanomaterial. Multiple coatings can be engineered on the IONP core to facilitate its application. (**c**) When the synthesized IONP interacts with physiological fluids, its properties can be altered. Referred to as the biological identity of the nanomaterial, this concept accounts for changes made to the colloidal status of the IONP after the formation of a biomolecule corona surrounding the IONP. This, in turn, can affect IONP endocytosis, toxicity, its detection by the immune system, or the signaling pathways that the IONP could trigger in cells with which it comes into contact.

Iron oxide nanoparticles (IONPs) belong to a family of inorganic nanomaterials that have increasingly become the focus of research over the last decade [1,2]. When used for targeted delivery of drugs, IONPs not only exhibit the advantages of nanoparticles such as their theranostic potential due to a high surface-to-volume ratio and surface-stemmed chemical labile residues that allow for chemical drug loading, but they also possess intrinsic superparamagnetic properties that permit magnetic targeting. The superparamagnetic phenomenon occurs when the size of certain magnetic materials is reduced below that of the single magnetic domain. For iron-based magnetic materials such as magnetite (Fe_3_O_4_) and maghemite (Fe_2_O_3_), magnetization is not retained after removal of the external magnetic field provided the core diameter does not exceed ~20 nm [3]. Magnetic properties such as these sparked interest in these nanoparticles, which was then translated into the most powerful non-invasive imaging technique available in clinical practice: magnetic resonance imaging (MRI) [4,5]. Furthermore, these superparamagnetic properties of IONPs also led to their application in magnetic field-driven tissue targeting. Indeed, these features make up the physical identity of IONPs, understood as the physical properties intrinsic to the metallic core (Table 1 and Figure 1). Physical identity ultimately determines the biomedical application of metallic nanoparticles in imaging/therapy, and the magnetic properties of IONPs make it possible for them to be used in MRI [5], magnetic targeting [6,7,8] and delivery [9,10], and hyperthermia [11,12,13].

The synthetic identity of a nanomaterial refers to the properties resulting from synthesis of the material. Synthetic identity encompasses not only the engineering of the core and surface coating but also the final shape and size of the nanoparticle. Nanomaterials are designed to have a synthetic identity that facilitates their intended application; for instance, ligands may be added to improve targeting (functionalization), and PEG may be included in the coating to increase circulation time. These physicochemical properties can therefore be manipulated to complement the physical identity of the nanomaterial and thus improve its functionality. As a result, nanoparticle surface modifications are key steps in developing nanoparticles for biomedical application. 

The concept of nanoparticle biological identity comes into consideration when the nanomaterial is exposed to the complex microenvironment of physiological fluids. This exposure triggers the formation of a corona composed of biomolecules, which can lead to changes in the aggregation state and alter the nanoparticle size. These complex interactions between the biological milieu and the nanoparticle depend on multiple factors such as synthetic identity, the biological microenvironment, and the interaction time with the organism. The possibility of functionalizing IONPs with biomolecules such as peptides [14,15], ligands [16], antibodies [17,18], aptamers [19,20], or RNAs [21] further enables IONPs to interact with a specific cell type or tissue; for instance, antibody-functionalized IONPs can specifically target antigen-expressing tumor cells, which allows local application of an alternative magnetic field for the induction of magnetic hyperthermia [22]. Understanding these interactions and how they influence the intended application of the synthesized nanoparticle is critical, as such knowledge not only allows for more rational nanomaterial design but could also enable these previously undiscovered characteristics to be harnessed for combinatorial therapies. 

As described above, both the synthetic and biological identities are integral to the nanoparticle surface (Figure 1) and together determine the utility of nanoparticles in biomedicine. Within biological systems, the identity of IONPs is therefore the result of effects that stem from their metallic core, their coating, and the interaction of this coating with the biological milieu. This review will attempt to summarize some of the common effects that IONPs display in biological systems due to their synthetic nature. Particular focus will be on how iron oxide influences biological processes, and how this effect, in turn, can alter myeloid, endothelial, and tumor-cell function.

## 2. Iron Oxide-Driven Biological Activities: Cellular Iron Metabolism and Reactive Oxygen Species 

IONPs are often deemed safe due to the existence of multiple pathways in the organism that can process the putative excess iron produced with nanomaterial injection. Yet the possibility of systemic toxicity caused by an excess of iron could constitute a major drawback for the clinical application of IONPs. The ramifications of surplus iron are of great importance, as the possibility of such repercussions in healthy cells will ultimately determine the efficiency of these nanoreagents for imaging/therapy. These biological effects are related to the physical identity of IONPs and thus may be common to all iron-based nanosystems. 

Based on the premise that IONP degradation products can represent an external source of iron for the cells, the first section of this review provides an overview of the cellular mechanisms driven by iron oxide in a physiological context. The discussion is focused on how iron oxide influences the activity of macrophages and endothelial cells, since these cell populations come into contact with the nanomaterial and are important regulators of iron metabolism in tissues. The influence of iron oxide on tumor cells is also addressed. Finally, the relationship between iron oxide and reactive oxygen species homeostasis is also presented, as it includes important mechanisms that govern the cellular response to IONPs. This section gives an overview of the multiple cellular pathways that could be affected by exposition to IONPs.

### 2.1. Cellular Components of Iron Metabolism: Macrophages 

**Figure 2:**
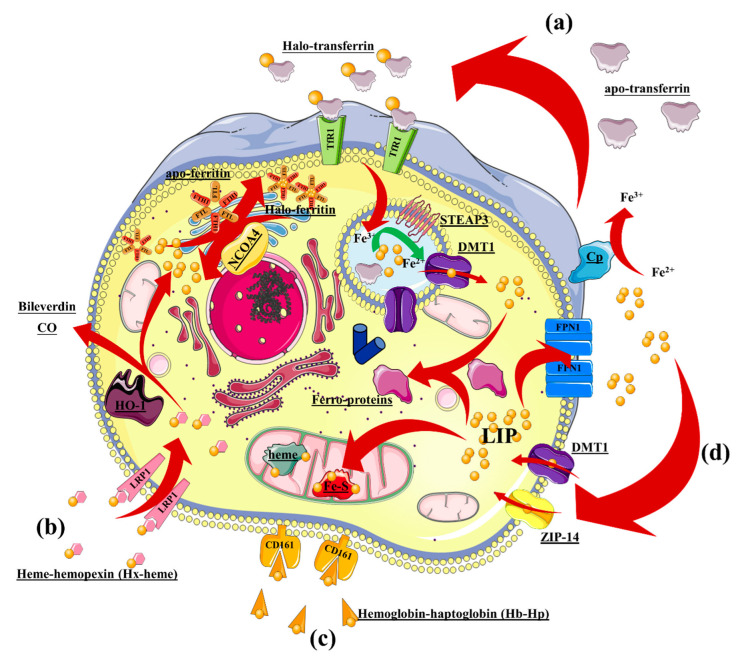
Overview of intracellular iron metabolism in macrophages. Iron intake is mediated by 4 major pathways: (**a**) Extracellular iron is scavenged by apo-transferrin into halo-transferrin, which is recognized be the transferrin receptor and internalized; (**b**) iron-containing heme–hemopexin is internalized after recognition by the LRP1 receptor; (**c**) iron-containing hemoglobin–haptoglobin is recognized by the CD161 receptor and internalized; and (**d**) non-Tf-bound iron ions (Fe^2+^) can be transported from the extracellular space into the cells by the transporters DMT1 and ZIP-14. CO, carbon monoxide; DMT-1, divalent metal transporter type 1; LIP, labile iron pool; LRP1, LDR-related receptor type 1; NCOA, nuclear receptor coactivator 4; STEAP3, six-transmembrane epithelial antigen of the prostate 3; ZIP-14, zinc transporter ZRT/IRT-like protein-14. Other iron sources can also contribute to the intracellular LIP, and these comprise dying erythrocytes and other cells phagocytosed by macrophages [37,38,39]. An additional source of intracellular iron is the result of the heme oxygenase-1 (HO-1) activity that splits heme–Fe (transported by LRP1) into Fe^2+^ and two anti-inflammatory mediators, i.e., biliverdin and carbon monoxide (CO) [40,41]. Both of these products of heme degradation exert an anti-inflammatory effect mediated by upregulation of IL-10, downregulation of pro-inflammatory cytokines such as TNFα and IL-6, and downmodulation of ROS production [42]. Besides these physiological sources contributing to the intracellular LIP, the degradation products of IONP cores can be considered as an external iron source that potentially augments intracellular iron content. It is thus important to understand how cells could cope with this excess iron.

The existence of physiological iron-recycling pathways makes IONPs highly biocompatible. Macrophages are key cells in systemic and tissue iron homeostasis [31]. Iron mobilization occurs systemically to support erythropoiesis [32] and bacteriostatic functions [33,34]. In the local environment, iron is mobilized from macrophages to tissues with iron need. This role relies on the metabolic machinery orchestrated by macrophages, which balances iron intake and export with their intracellular iron pool. Iron intake is mediated by four main pathways (Figure 2): (1) Extracellular iron is scavenged by apo-transferrin (apo-Tf), resulting in halo-transferrin (halo-Tf), which is recognized by the transferrin receptor (TfR1) and then endocytosed in clathrin-dependent vesicles; (2) iron-containing heme-hemopoxin interacts with the LDR-related receptor (LRP1), which is consequently endocytosed; (3) iron-containing hemoglobin–haptoglobin is recognized by the hemoglobin–haptoglobin receptor (CD161) and internalized; and (4) non-Tf-bound Fe^2+^ is imported by the membrane-localized transporters DMT1 (divalent metal transporter) and ZLP14 (zinc transporter ZRT/IRT-like protein-14). Imported iron then enlarges the intracellular labile iron pool (LIP) destined for trafficking, storage, and/or export through ferroportin-1 (FPN1) [35,36]. 

Most of the LIP is delivered to the mitochondrion in which Fe^2+^, as part of hemes and Fe–S clusters within enzymes, assists the electron-transfer cascade and enzymatic activity. Iron cations that are neither trafficked into mitochondria nor exported by membrane transporter FPN1 [43] are stored by ferritin heteropolymers composed of ferritin heavy (FTH1) and ferritin light (FTL) chains. This heteropolymer can cage up to 4500 iron atoms [44]. This complex is disassembled and degraded by nuclear receptor coactivator 4 (NCOA4) when the LIP is low, releasing iron into the cytoplasm in a process termed “ferritinophagy” [45,46]. Exported Fe^2+^ can be converted into Fe^3+^ by ferroxidase enzyme ceruloplasmin (Cp), which is located extracellularly; this is a necessary step for the loading of iron on apo-transferrin [47]. 

### 2.2. Cellular Components of Iron Metabolism: Endothelial Cells 

In addition to macrophages, endothelial cells also display a trafficking mechanism that supplies iron to the local tissue environment. This is evidenced by the high density (~100,000 per cell) of TfR found in brain microvascular endothelial cells (BMECs), which enables these cells to sequester iron [48]. The entry of iron in human BMECs does not solely depend on TfR–Tf clathrin-dependent internalization, which accounts for ~50% of the total iron internalized; rather, BMECs also rely on the entry of non-Tf bound iron (NTBI) species mediated by cytoplasmic membrane-associated DMT1 assisted by transmembrane reductases [49]. This makes endothelial cells critical regulators of iron transport through the blood–brain barrier (BBB) [50]. It should be noted that an increase in intracellular Fe^2+^ can be detrimental to proper BMEC functioning as exemplified by the appearance of secondary brain injuries after intracranial hemorrhage due to the excess of iron-containing factors, such as hemoglobulin, that are released during injury [51,52]. This detrimental effect is related to the induction of reactive oxygen species (ROS) as found by Katsu et al. [53], which is discussed in a subsequent section of this review. 

Once Tf-associated Fe^3+^ reaches the endosomal compartment, it is reduced to Fe^2+^ by a cytosolic donor such as NAD(P)H and catalyzed by an endosomal ferrireductase, which in BMECs include duodenal cytochrome b (Dcytb) and the six transmembrane epithelial antigen of prostate 2 (STEAP2) (Figure 2; [49,54]). The importance of ECs in iron homeostasis is not only associated with their barrier function; these cells also act as a sensor and play a supportive role that promotes iron accumulation in bystander cells. Indeed, Canali et al. and Koch et al. independently found that liver sinusoidal endothelial cells that internalize Fe^2+^–Tf–TfR secrete bone morphogenic protein 2 and 6 (BMP2/6), which in turn downmodulate hepcidin expression by bystander hepatocytes. If this loop is inhibited, overloading of iron occurs, leading to a pathological condition similar to the hemochromatosis phenotype [55,56]. Therefore, ECs are an important regulator of iron homeostasis that connect iron uptake (enterocytes), trafficking/recycling (macrophages), and storage (hepatocytes/erythrocytes) by transporting iron from circulation toward tissue and modulating iron storage in bystander cells. 

### 2.3. Iron Homeostasis and Cancer Cells

In cancer cells, iron homeostasis is often dysregulated, thus becoming a hallmark for cancer initiation and progression. Since Richmond [57] first described the induction of sarcoma upon repetitive intramuscular administration of an iron dextran complex, several reports demonstrated that iron can promote carcinogenesis [58,59,60,61]. This tumorigenic property of iron appears to reflect underlying DNA damage induced by oxidative stress [62,63]. 

Beyond the tumorigenic potential of iron, tumor cells also exhibit a shift in iron homeostasis toward exacerbated intracellular iron sequestration through increasing uptake and storage, downmodulating export, or both mechanisms. This is evidenced by the downmodulation of FPN1 and overexpression of its natural inhibitor, hepcidin, in a variety of solid tumors, e.g., breast, ovarian, and prostate cancer [64,65,66,67,68]. When disturbed, the ferroportin–hepcidin axis indeed promotes breast-tumor growth mediated by BMP6- and IL-6-induced hepatic hepcidin, thus leading to an increase in the intracellular LIP [69]. The involvement of the hepcidin-ferroportin-1 axis in cancer progression has been associated not only with tumor growth but also with metastasis [70]. Epigenetic regulation mechanisms also contribute to cancer-associated iron accumulation, including microRNA-mediated downmodulation of ferroportin [71,72], mTOR complex 2-mediated regulation of iron-related genes via acetylation of histone 3 [73], and hypermethylation of DNA promoter [74,75]. The capacity of tumor cells to metabolize iron, therefore, requires consideration when developing IONP-based therapeutic strategies for cancer.

### 2.4. Iron Oxide and Redox Homeostasis

Part of the effects that IONP exert on cellular function depends on the iron cation released from the NP iron cores. Iron cations engage in cellular iron metabolism machinery and likely disturb redox homeostasis. Reduced iron is a strong producer of reactive oxygen species (ROS) through the Fenton reaction, whereby the electron-donor Fe^2+^ cation drives hydrogen peroxide (H_2_O_2_) to split into hydroxyl anion (HO^−^) and the more reactive hydroxyl radical (•OH), which is able to react with biomolecules at a diffusion-controlled rate (Equation (1) and [76,77]). The Fenton reaction remains the consensus explanation for the underlying mechanism that results in intracellular ROS production by iron oxide nanoparticles, which in turn sustains hydroxyl radical production from H_2_O_2_ and potentially disturbs redox homeostasis.

Fe^2+^ + H_2_O_2_ → Fe^3+^ + •OH + HO^−^ (1)

**Figure 3:**
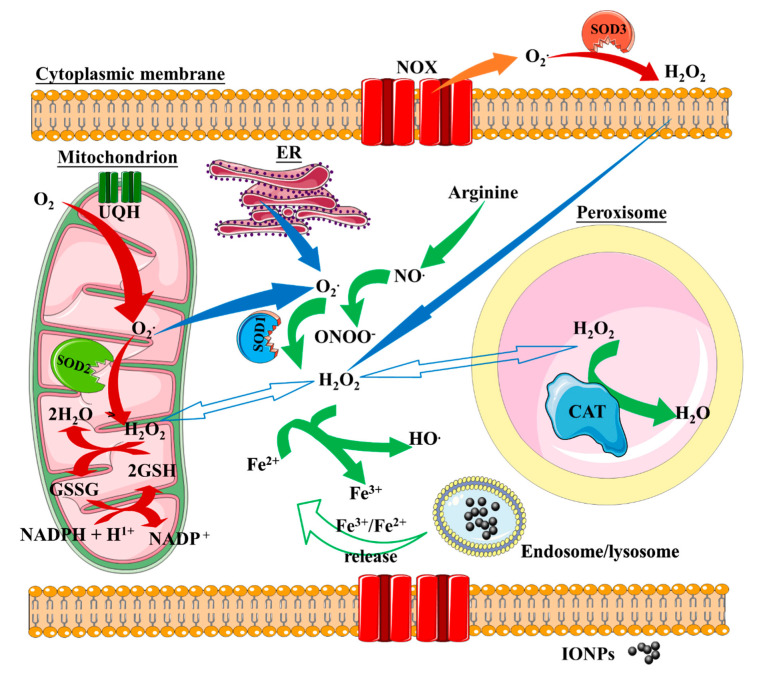
Overview of intracellular ROS metabolism and involvement of iron through the Fenton reaction. H_2_O_2_ arises from intracellular sources via the activity of cytoplasmic SOD1, mitochondrial SOD2, and peroxisome molecular machinery. Extracellular H_2_O_2_ is generated by extracellular SOD3. H_2_O_2_ production is central to the production of ROS by iron cations through the Fenton reaction. Iron cations released from IONP cores drive H_2_O_2_ to split into hydroxyl anion (HO^−^) and the more reactive hydroxyl radical (•OH). SOD, superoxide dismutase; CAT, catalase; UQH, reduced ubiquinone; GSH and GSSG, reduced and oxidized glutathione, respectively; NOX, NADPH oxidase; ER, endoplasmic reticulum.

Redox homeostasis is maintained by a variety of enzymatic reactions that balance the production of intracellular H_2_O_2_ with antioxidant responses (Figure 3). The vast majority of H_2_O_2_ arises from intracellular sources, i.e., mitochondrial superoxide dismutase (SOD2) [78], peroxisome molecular machinery (organelle associated with lipid metabolism, [79,80]), and cytoplasmic superoxide dismutase 1 (SOD1) [81]. The main extracellular source of H_2_O_2_ is derived from the activity of superoxide dismutase 3 (SOD3) [82]. 

**Figure 4:**
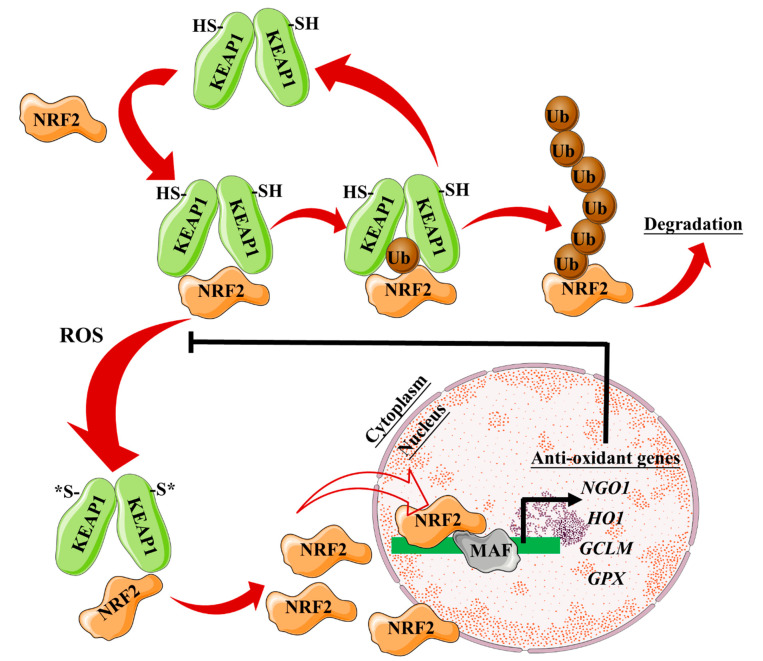
Overview of the ROS/Nrf2-axis and its anti-oxidant effect. Kelch-like ECH-associated protein (KEAP1) homodimers promote NRF2 ubiquitylation leading to proteasomal degradation. KEAP1 is then recycled to bind newly synthesized NRF2. Oxidative stress drives oxidation of key cysteine residues on KEAP1, preventing NRF2 ubiquitylation. NRF2 can thus accumulate and translocate to the nucleus, where it dimerizes with one of the small MAF proteins to promote the transcription of anti-oxidant genes. GCLM, glutamatecysteine ligase modifier subunit; GPX, glutathione peroxidase; KEAP1, Kelch-like ECH-associated protein 1; MAF, musculoaponeurotic fibrosarcoma; NRF2, nuclear factor (erythroid-derived 2)-like 2.

As a consequence of H_2_O_2_ production, a variety of cellular anti-oxidant responses are triggered to overcome the oxidative stress and protect cellular functions, including activation of nuclear factor (erythroid-derived 2)-like 2 (Nrf2) and heme oxygenase-1 (HO-1) [83,84]. Nrf2 is a transcription factor involved in the antioxidant response element (ARE)-mediated transactivation of anti-oxidant enzymes, e.g., HO-1 and NQO-1, which reestablishes the correct cellular redox balance. Upon oxidative stress or phosphorylation by protein kinases, Nrf2 is released from its cytoplasmatic repressor, Kelch-like ECH-associated protein 1 (Keap1), and translocates to the nucleus where it interacts with ARE regions within the promoter of anti-oxidant genes [85,86] (Figure 4). 

**Figure 5:**
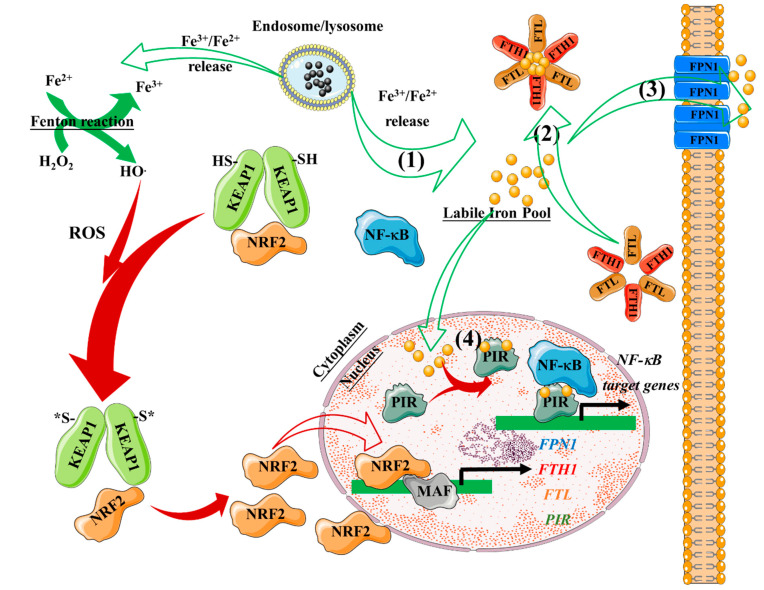
The ROS/Nrf2-axis regulates the labile iron pool (LIP). Iron cations are released from the endosome/lysosome (**1**) and Fenton reaction-induced ROS triggers NRF2 translocation to the nucleus and promotes the expression of iron metabolism-associated genes (ferroportin-1-*FPN1-*, ferritin heavy chain-*FTH1-*, ferritin light chain-*FTL-*, and Pirin-*PIR-*). (**2**) Labile iron can be sequestered in ferritin, which comprises FTL and FTH1 subunits. (**3**) Labile iron can be exported out of the cytosol by ferroportin-1. (**4**) Labile iron can bind to PIR and influence NF-κB transcriptional activity. NRF2, nuclear factor (erythroid-derived 2)-like 2; MAF, musculoaponeurotic fibrosarcoma.

NRF2 translocation to the nucleus also promotes the transcription of iron metabolism-associated genes (e.g., ferroportin-1, ferritin heavy chain, ferritin light chain, Pirin). The cell thus triggers a gene transcription program that can mitigate ROS production due to the presence of Fe^2+^ cations. Among the genes regulated by NRF2, Pirin is an Fe-binding protein that can regulate the activity of NF-κB, a master regulator of proinflammatory responses that controls immune and stress responses (Figure 5). Pirin appears to act as a sensor of the cell redox status, which allows the NF-κB transcription factor to engage in DNA binding and is thought to promote NF-κB-dependent responses to oxidative stress [87]. Iron cations can, therefore, alter cellular redox homeostasis through several mechanisms. Iron cations promote ROS production. Moreover, they can directly affect the NF-κB transcription program, likely through their interaction with Pirin. Iron cations released from IONP cores thus have the potential to modulate ROS production and modify immune and stress responses. IONP effects on these pathways could be exploited to enhance the bioactivity of nanoreagents.

## 3. Iron Oxide Nanoparticle Biodegradation

Having summarized the complex molecular and functional machinery driven by labile iron and ROS, it becomes apparent that understanding IONP degradation is key to evaluating the biological identity of IONPs. IONP degradation by-products can become an external source for intracellular iron and ROS and therefore can modify cell responses. This section of the review provides an overview of IONP biodegradation, and how this defines their biological identity. IONP degradation is dependent on multiple factors, among which we mainly focus on the effects of the protein corona, the endocytosis routes, and the cellular degradation machinery. These factors define how IONPs are perceived in a biological system and consequently how the biological system reacts to the IONPs.

### 3.1. IONP Biodegradation and Biological Identity 

Long term biodegradation studies have identified a loss in magnetic properties of IONPs when inoculated [88], which correlates with a concomitant increase in iron metabolic routes consistent with a degradation process [89]. IONP-derived iron availability depends on the endocytosis mechanisms by which the nanoparticles enter the cells and how this internalization modulates their degradation and the release of iron into the cytoplasm. The harsh environment found in phagolysosomes (low pH of 4.5–5), high ionic strength, and the presence of a variety of degrading enzymes such as acid hydrolases and cathepsins is mainly responsible for nanomaterial degradation [90,91,92,93]. From a structural point of view, IONP degradation encompasses the targeting of three discernible physicochemical and biochemical entities: (1) the enzymatic degradation of the protein corona that usually surrounds the nanoparticle when it comes into contact with physiological fluids, and which determines how IONPs interact with biological systems (biological identity); (2) degradation of the engineered surface coating (synthetic identity); and (3) the disintegration of the metallic core (physical identity). Each step is influenced by the nature of the material to be degraded, the cell type, and cellular metabolic status. 

### 3.2. IONP Degradation and Protein Corona

It is nowadays accepted that IONPs adsorb a plethora of ions and biological molecules (i.e., lipids, sugars, proteins) when they come into contact with biological fluids such as blood [94]. This biomolecular corona ultimately affects the interaction of non-functionalized IONPs with biological systems, a property known as “biological identity”. The biomolecular corona must be taken into account when considering the intrinsic biological effects that IONPs can produce. Indeed, Escamilla-Rivera et al. found that PEG-coated IONPs absorb a wide variety of complement recognition proteins when incubated with human serum, leading to complement activation when injected in mice [95]. Similarly, Zhu et al. demonstrated that the addition of IONPs to the hyaluronic acid (HA) scaffold enhances osteogenesis in vivo, most likely mediated by a dynamic formation of a protein corona enriched in complement-, wound healing-, and inflammatory-related factors [96]. These reports thus suggest that the protein corona can endow IONPs with immunomodulating features. However, the biomolecule corona largely depends on the surface coating of the metallic core of IONPs as demonstrated by Vogt et al. These authors found that silica-coated and dextran-coated IONPs adsorb a distinctive subset of serum proteins, which leads to a differential effect on primary human macrophages [97]. Furthermore, the inherent dynamic nature of the protein corona, more specifically the so-called soft corona that exhibits a high exchange rate, can affect the biological identity of IONPs. Ashby et al. reported that a highly hydrophobic surface coating promoted the deposition of human serum proteins having larger hydrophobic domains and a much faster exchange rate, and thus a more dynamic protein corona. A similar correlation was found between larger core diameters and protein corona dynamics [98]. Nanoparticle size also seems to correlate inversely with the protein corona, not only in terms of composition but also in protein amount, due to a curvature effect [99]. Nonetheless, Ashby et al. evidenced for the first time that a soft corona is much less influential in IONP endocytosis than a hard corona [98]. This was demonstrated by pre-incubating amphiphilic block copolymer (AMP)-coated IONPs of 10 and 25 nm with transferrin and assessing their endocytosis in a macrophage model. Even when non-functionalized IONPs showed a similar internalization rate, 10-nm AMP-coated IONPs underwent increased endocytosis by macrophages when pre-incubated with transferrin as compared to 25-nm AMP-coated IONPs, suggesting that the presence of a hard corona is a prominent factor in receptor-mediated endocytosis. 

Synthetic identity has a clear influence on biological identity, i.e., how the composition of the adsorbed protein corona is determined by the nature of the surface coating (Figure 1 and Table 2). Stepien et al. proved that two different surface coatings, i.e., glucose and polyethylene glycol (PEG), promote the formation of protein corona with a distinct composition. These synthetic differences also impacted the IONP degradation rate in vitro, as glucose-coated IONP degradation appeared to be accelerated. However, in vivo assessment of biodegradation showed contrasting results, with PEG-coated IONPs exhibiting faster biodegradation and clearance [100]. The influence of synthetic identity on the composition of a protein corona and the biodegradation rate also leads to another important question, i.e., how these complex interactions determine nanoparticle toxicity. Ma et al. revealed that the nature of a corona protein determines the biodegradation rate and, consequently, cell toxicity; the authors did this by comparing nanoparticles with three different coronae: human serum albumin (HSA), *γ*-globulin, and serum fibrinogen. In their experiments, nanoparticles with HSA-composed corona degraded faster, and as a result, cell viability decreased concomitantly with a reduction in ATP production and mitochondrial membrane potential [101]. Lu et al. further found that by varying the chemistry of the synthetic identity, it is possible to tailor the way proteins and NPs interact [102]. Protein–NP interactions were found to depend on hydroxyl group availability. HSA/IgE interaction with the NP graphene/gold surface correlated inversely with available hydroxyl groups. ApoE interaction with these NPs was less dependent on these groups, which likely prolonged the circulation of these ApoE-rich corona NPs when compared to their IgE-rich counterparts. Liu et al. demonstrated that external physical cues are yet other factors that affect the protein corona composition of IONPs [103]. When incubated in vitro with DMEM medium with 10% FBS, the protein corona deposited on glutamine-coated IONPs decreased drastically in terms of protein amount and composition when an external static magnetic field (SMF) was applied. This discovery has a direct consequence in IONP-based magnetic targeting and magnetic hyperthermia, as these data indicate that the application of the magnetic field can affect the biological identity of the IONPs. Indeed, the SMF-adjusted protein corona diminished the immunological response driven by IONPs as measured by the quantity of secreted cytokines by IONP-treated macrophages. These studies illustrate how interlinked the synthetic and biological identities of NPs are and how this will affect the intrinsic biological activities of IONPs. This is currently an active field of investigation in which our laboratory seeks to understand how different coatings influence protein corona composition and, ultimately, affect IONP degradation.

### 3.3. Endocytosis and IONP Degradation

The endocytic mechanisms of IONPs are often complex and involve several pathways (Table 3). Such complexity arises from the impact of structural factors such as IONP size, charge, surface coating, and the “biological identity”, while the cell type profoundly delineates the endocytic processes that can take place. The protein corona also influences how IONPs are endocytosed by cells, which in turn determines the NP intracellular fate [106,107,108]. Endocytosis can occur through two main pathways in mammalian cells: (1) pinocytosis, a process that mediates the internalization of fluids and small molecules within small vesicles (<0.15 μm) and comprises macropinocytosis, clathrin-, caveolin-dependent, and caveolin-independent endocytosis [109,110,111]; and (2) phagocytosis, a process that involves the ingestion of larger particles, e.g., microorganisms and cell debris, via larger intracellular vesicles called phagosomes (>0.25 μm) [112,113,114]. Most of the pinocytic processes are receptor-dependent, and there are key differences among them that can affect the intracellular fate of IONPs. For instance, while caveolin-dependent endocytosis requires membrane invagination around cholesterol-rich rafts, thus causing it to be a slow mechanism, clathrin-dependent pinocytosis is often a fast process connected to internalization of nutrients such as iron-laden transferrin [115]. Rezai et al. demonstrated that PVA–PLGA-NPs are internalized differently depending on dysopsonin/opsonin abundance in the protein corona. Higher opsonin proportion favored FcR-dependent internalization, while in FcR^–^ cells, opsonins hampered nanoparticle internalization [27]. This ratio between dysopsonin and opsonin could, therefore, be key not only for prolonging NP circulation, but could also be manipulated to improve targeting towards a particular cell type or even a subcellular compartment.

Nonetheless, synthetic identity can also impact the nanoparticle uptake as elucidated by Feng et al. [116], who compared the internalization of polyethyleneimine-coated IONPs and PEGylated counterparts. In line with our results obtained with PEI-coated IONPs, these researchers found that highly positive-charged IONPs are taken up at a higher rate by RAW264.7 cells as compared to the nearly neutral-charged PEGylated IONPs. This profound influence of surface charges on nanoparticle endocytosis has been addressed before [117], indicating that positively charged IONPs tend to accumulate intracellularly more than their negatively charged counterparts [118], and surface charge is directly related to the biological effects on cells [119]. More importantly, negatively charged IONPs appear to accumulate first in endosomes and later in lysosomes, while positively charged IONPs (e.g., PEI-coated) seem to accumulate largely in lysosomes [118]. Such behavior can indeed influence the intracellular degradation rate of nanoparticles.

### 3.4. IONP Biodegradation by Cellular Machinery

Once internalized, IONPs can be degraded by cellular machinery. Ferritin plays a crucial role in protecting cells from ROS-triggered injury upon IONP internalization by storing excess Fe^3+^ derived from the nanoparticles. An increase in ferritin levels after IONP treatment was detected in vitro in several macrophage models and in vivo in liver extracts, confirming that this iron metabolic pathway is activated by IONP administration [89]. In vivo experiments by Maraloiu et al. reveal that maghemite nanoparticles are degraded down to the non-toxic complex ferritin in spleen and atherosclerosis plaques [134]. Systemically, IONP biodegradation starts with the capture of IONP as early as 3 h post-intravenous injection, mainly by endothelial cells present in liver sinusoids and spleen capillary as well as by Kupffer cells and macrophages in the liver and spleen. Twenty-eight days later, most EC-associated IONP clusters disappear, and the remnants exhibit high-density packaging along with an increment in ferritin deposits as visualized by the presence of less-dense clusters [135]. A study by Mejías et al. presented some of the earliest evidence that the physical identity of IONPs changes once they enter the body. They observed that the magnetic susceptibility of organs such as liver and spleen exhibited an acute increase after 30 min due to IONP accumulation, but diminished thereafter. This change reflected a shift in iron-core status from a superparamagnetic core to a non-superparamagnetic form [88]. Similar behavior was observed in rats, where DMSA-coated IONPs demonstrated a faster degradation rate as compared to PEG-coated IONPs, suggesting that synthetic identity impacts systemic biodegradation [136]. Mazuel et al. studied IONP degradation within stem cells by measuring the magnetic properties of IONP-loaded spheroids. The magnetic properties (magnetometry and magnetophoresis) of human stem-cell spheroids varied over time (up to 27 days), evidencing no changes in the total amount of intracellular iron. In cellulo IONP biodegradation was evidenced by IONP structure loss and the appearance of ferritin cages near iron-loaded endosomes. The extensive collapse of spheroid magnetism was accompanied by demagnetization at the single-endosome level [92], confirming the shift towards non-superparamagnetic iron forms during biodegradation. Using a similar system, Curcio et al. studied the intracellular biodegradation of magnetosomes, a magnetite-based particle biosynthesized by *Magnetospirillum magneticum*. The authors found that by measuring cellular sample magnetization, human stem cells gradually degraded the magnetosome material into ferrihydrite within their lysosomal/endosomal compartment over 21 days [137]. As a consequence, photothermal conversion of stem-cell spheroids was lowered over time. Curiously, Curcio et al. also found that when magnetosome-loaded stem cells are cultured in 2-D, magnetization decreased by the third day of culture but recovered after 21 days, suggesting a de novo biosynthesis of intracellular magnetic nanoparticles, most likely out of the magnetosome degradation-derived iron pool [137]. Electron-microscopic evidence of IONP presence in cellular degradative compartments after internalization and changes in magnetization have thus established that IONPs are likely degraded intracellularly in lysosomal/acidic compartments and that this results in a loss of physical properties in the nanomaterial over time. However, studies have shown disparities in the timeframe of these events, which could be due to differences in the nanomaterials employed.

It is reasonable to think that for IONP cores to undergo massive degradation, not only their protein corona must be degraded but also their synthetic identity, i.e., surface coating. However, only recently have reports begun to address such matters. Zhu et al. demonstrated that poly-(isobutylene-alt-maleic anhydride)-graft-dodecyl (PMA)@IONPs, onto which three different dyes were covalently attached by amide bonds, are susceptible to amide bond cleavage by fetal bovine serum, aminotransferase (AST), and trypsin [138]. Similarly, Sée et al. demonstrated that a peptide monolayer deposited onto a gold nanoparticle undergoes enzymatic degradation by cathepsin L when nanoparticles are endocytosed by HeLa cells [139]. Carboxydextran coating on IONPs also undergoes lysosomal degradation by a-glucosidase upon internalization by macrophages, as demonstrated by Lunov et al. [93]. This suggests that multiple enzymatic activities can participate in protein corona and surface-coating degradation, which could in turn affect the kinetics of degradation of the IONP core. Indeed, our group is currently focusing on understanding which lysosomal enzymes can contribute to the degradation of IONPs with different coatings. 

## 4. IONP Effects is Dependent on Cell Type and Status 

We have discussed thus far how IONP effects depend on the biological pathways they activate when they are internalized and degraded. These are not the sole factors that govern their biological effects. The cell type that the IONPs encounter also dictates their effects in biological systems. In the following sections, we summarize how IONPs can affect the biology of cells they are likely to interact with in an antitumor therapeutic setting. IONPs interact with myeloid cells specialized in the capture of particulate materials, such as macrophages or dendritic cells. As IONPs circulate through the vascular system, they also encounter endothelial cells. Finally, IONP also affect the tumor microenvironment. The following section provide an overview of how the presence of iron can affect these cell types. We also discuss the possibility of using these intrinsic biological properties of IONPs to enhance their activity in a therapeutic setting. 

### 4.1. IONPs and Myeloid Cells

#### 4.1.1. Iron Metabolism and Macrophage Polarization 

The interactions and effects of metallic nanoparticles on macrophage activation have been concerns in terms of nanomaterial imaging/therapeutic efficacy and systemic nanotoxicity [140]. Iron oxide nanoparticles are among the most widely used nanomaterials, even in clinical settings, and thus the study of how they interact with myeloid cells is of great importance for researchers and clinicians. The relevance of iron in myeloid cells is exemplified by the involvement of this redox-active metal in several essential enzymes and protein regulators, all classified as hemoproteins, which participate in key cellular processes for macrophage activity in inflammation (e.g., NADPH oxidase 2, cyclooxygenases 1 and 2, inducible nitric oxide synthase). Macrophages are also central to the systemic trafficking of iron [141,142]. Splenic marginal metallophilic macrophages phagocytose senescent erythrocytes and release heme-derived iron back into circulation to support different systemic and local functions such as pro-inflammatory and bacteriostatic response [34,143]. Macrophages retain iron during inflammation as a result of the binding of the acute-phase protein, hepcidin, which mediates an increase in iron uptake and the internalization and degradation of the iron export transporter of the heme-free iron ferroportin [144,145]. A more detailed review of iron macrophages has been published elsewhere [43]. 

**Figure 6:**
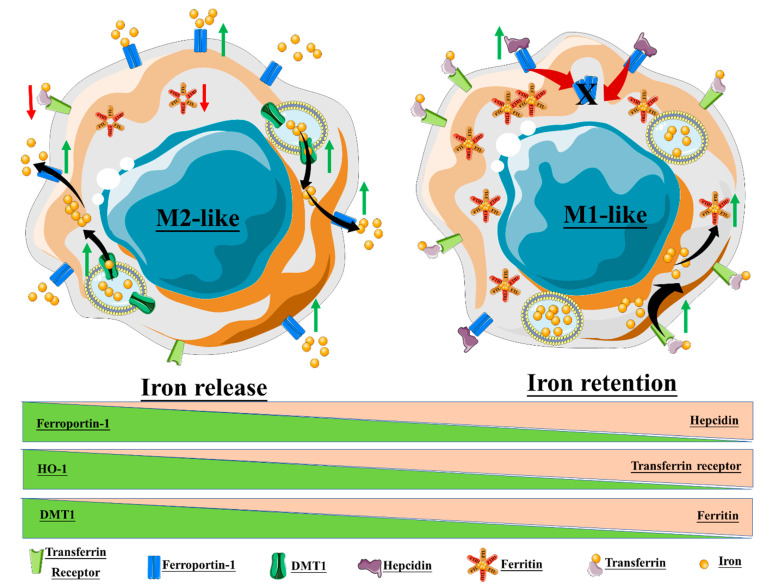
Macrophage polarization and iron homeostasis. The M2-like phenotype exhibits high expression of ferroportin-1, the divalent metal transporter-1 (DMT1), and heme oxygenase-1 (HO-1), thus promoting a state of iron release. On the contrary, the M1-like phenotype promotes intracellular iron retention through the upregulation of ferritin, hepcidin, and the transferrin receptor.

The macrophage response to iron is also affected by polarization, and in turn, iron can affect macrophage polarization (Figure 6). Recalcati et al. found that M2 macrophages express higher amounts of iron metabolism-related proteins, e.g., transferrin receptor (TfR1), iron-responsive proteins (IRP), and ferroportin (Fpn), when compared to unpolarized cells, while M1 macrophages downregulated these proteins. The M1 phenotype also endows macrophages with iron sequestration ability, while the M2 phenotype promotes iron release [146]. Exogenous iron can promote macrophage polarization toward an M1 phenotype through the production of ROS and, in consequence, enhance p300/CBP acyltransferase activity and the acetylation of p53 [147]. Intracellular iron also plays a crucial role in the M2-/M1-balance according to Agoro et al. Administration of an iron-rich diet in mice promoted the in vivo expression of high levels of the M2 markers *Arg1* and Ym1 in liver and peritoneal macrophages. More interestingly, an iron-rich diet prevented the mice from developing an LPS-induced inflammatory response through an M1–M2 reversion, while iron deficiency exacerbates the endotoxin-induced inflammatory response [148]. The latter finding was also corroborated by Pagani et al. when studying the response of iron-deprived mice to LPS challenge. These authors found that iron-deprived hepatic and splenic macrophages expressed higher levels of IL-6 and TNF than those of healthy mice, indicating that iron content negatively regulates M1 response [149]. Nonetheless, the role of iron in the M1–M2 balance is not consistent throughout the literature (Table 4). Hoeft et al. observed that iron overloading aggravates LPS-induced inflammatory response in mice, most likely mediated by an increment in mitochondria biogenesis in iron-loaded macrophages [150]. An unrestrained M1 response has been described for iron overloading in chronic inflammatory diseases, such as atherosclerosis and chronic venous leg ulcers, through overproduction of hydroxyl radicals and TNFα [151]. Likewise, iron load appears to promote a persistent M1 macrophage population in the injured spinal cord which, in addition to TNFα expression, prevents the injury site from being properly repaired [152]. Due to the multiple effects of iron on macrophages, IONP degradation products therefore have the potential to alter macrophage polarization.

#### 4.1.2. IONP Recognition by Macrophages and Activation

It has been proven that toll-like receptors mediate most macrophage reactions to iron oxide nanoparticles. We have demonstrated that polyethyleneimine-coated IONPs trigger macrophage activation, partially through TLR-4 engagement and production of ROS [121]. Autophagy is often activated upon nanoparticle phagocytosis, as demonstrated by Jin et al., who studied the effect of two FDA-approved iron-oxide nanoparticles, resovist and ferumoxytol [158]. The macrophage-like cells RAW 264.7, enclosed iron oxide nanoparticles within the endosome, early autophagic vacuole and eventually double-membrane autophagic vacuoles that contained nanoparticles, small internal vesicles, and cellular and membrane debris. These structural changes were accompanied by the formation of LC3 puncta and overexpression of sequestosome identified by p62/SQSTM1, an autophagy receptor that links ubiquitinated proteins and organelles with autophagosomes [164,165,166]. Noticeably, IONP-induced autophagy was mediated by the activation of the TLR4-p38-Nrf2 pathway rather than the classical autophagy machinery dependent on ATG5/12, as pre-treatment with the TLR4 signaling inhibitor, CLI-095, prevented IONP-loaded macrophages from exhibiting autophagic activities [158]. 

Another key issue for re-programmed macrophage-based therapies is related to interference by iron oxide nanoparticles with the adequate differentiation of monocytes into mature and competent macrophages. Vallegas et al. found out that poly(acrylic acid)-coated IONPs do not alter the viability of monocyte-derived macrophages during differentiation, but inhibit the secretion of LPS-induced cytokines such as IL1β, IL-6, and IL-10 [167]. Nonetheless, Dalzon et al. found that iron oxide carboxymaltose nanoparticles, known as FERINJECT®, do not significantly modulate LPS-induced cytokine profile in primary macrophages or hamper their ability to migrate towards a chemotactic stimulus, suggesting a clear dependence on IONP nature for macrophage activation status [168]. A clearer effect of the IONP on myeloid cells was described by Xu et al., who observed that ferumoxytol inhibits the suppressing functions of myeloid-derived suppressor cells (MDSCs) [169]. Contrasting with most reports in which IONPs were shown to act as ROS inductors, ferumoxytol treatment caused a ROS reduction in MDSCs, as evidenced by the decrease of the p47phox component of the nicotinamide adenine dinucleotide phosphate–oxidase (NOX) complex responsible for ROS production in MDSCs. Furthermore, ferumoxytol promotes bone marrow-derived MDSC differentiation into macrophages, reducing the appearance of these cells during sepsis-like scenarios. As a result, ferumoxytol ameliorates LPS-induced sepsis in mice [169]. 

It is equally important to understand how the different macrophage populations respond to IONP treatment, particularly as concerns approaches intended for imaging of MRI-visible macrophages, e.g., inflamed sites [170,171]. Each macrophage phenotype indeed expresses different factors involved in iron metabolism, and thus exhibits divergent iron sensitivity (Figure 5 and [146]). Zini et al. demonstrated that M2-polarized THP1 macrophages internalized significantly more IONPs than M1-polarized and M0, leading to a higher T1 signal in M2 macrophages and a higher T2* signal in M0 macrophages [172]. Internalized IONPs could also, in turn, exert effects on polarization and iron metabolism. In one example, our group showed that DMSA-, APS-, and aminodextran-coated IONPs changed iron metabolism towards an iron-sequestering status in M2-like macrophage [163].

Zhao et al. elegantly demonstrated that the FDA-approved iron oxide nanoparticle, ferumoxytol, synergizes with the TLR3 agonist poly (I:C) in inducing macrophage activation, thereby exerting a potent anti-tumor effect in a melanoma model [173]. Noticeably, the effect observed by these authors comprised cell contact-dependent and -independent molecular cues mostly triggered by ROS burst and phagocytosis of tumor cells in vitro. The synergistic effects of poly (I:C) and ferumoxytol treatment in vivo impaired primary B16F10 tumor growth, and subsequent lung metastasis appearance more efficiently than either treatment alone. This reduction in tumor growth correlated with an increase in pro-inflammatory macrophages within the tumor nest. More recently, Wang et al. demonstrated that ferumoxytol is primarily internalized by macrophages through scavenger receptors, i.e., SRI/II, and not mediated by complement C3b, as these rather large nanoparticles (30 nm) do not exhibit C3b deposition on their surface [174]. Taking another approach, Wang et al. proved that the intracellular TLR9-agonists CpG and ferumoxytol also synergize to promote an M1-like phenotype in macrophages with anti-tumoricidal capacity [175]. While the results outlined thus far are mainly focused on the synergy between IONPs and TLR agonists, others have demonstrated that the pro-M1/anti-tumor properties of ferumoxytol are intrinsic to the NP. Zanganeh et al. showed that the ferumoxytol-loaded macrophage-like cells, RAW264.7, induce apoptosis in MMTV-PyMT cancer cells mediated by Fenton reactions [176], leading to retardation in tumor growth in vivo. More importantly, intravenous pre-treatment with ferumoxytol protected mouse liver from KP1 tumor-cell infiltration, and this was associated with an M1-like phenotype of infiltrating macrophages and a loss of M2-like features in resident macrophages [176]. 

While studying the artificial reprogramming of macrophages for cancer cell therapy, Li Chu-Xin et al. found that feeding macrophages with hyaluronic acid-modified iron oxide NPs (HION) or bare iron oxide NPs (ION) triggered consistent production of ROS and pro-inflammatory cytokines [177]. Consequently, both HION-fed and ION-fed macrophages exerted an anti-tumor effect on the murine breast-tumor cell line 4T1 in a cell contact-independent manner by inducing active caspase 3 and inhibiting cell proliferation. The tumor microenvironment is known for its highly immunosuppressive profile, which comprises M2 macrophage populations that sustain tumor growth while hindering a pro-inflammatory shift [178]. This M2 macrophage population is believed to arise from resident macrophages and bone marrow-derived monocytes engaged in M2 programming by tumor cell-derived factors such as IL-10. Therefore, it is desirable that macrophage-based antitumor therapy not only induces an M1 phenotype from naive macrophages, but also that it reverses the resident M2 program into an M1 phenotype. In a related study, Chu-Xin et al. found that HION-loading provided M1 macrophages with resistance to M2-inducing factors and triggered M2-to-M1 reversion [177]. The in vivo tumor tropism of HION also provoked a reduction in tumor growth that was most likely due to decreased proliferation and apoptosis rates, thus indicating that this nanoreagent could be used to directly affect tumor cell growth and/or be employed for macrophage reprogramming in the tumor microenvironment. 

Given the impact that IONPs can have on macrophage activation, it is only logical to exploit this intrinsic activity to potentiate antigen-specific immune responses by targeting the antigen-presenting capacity of myeloid cells. Based on this reasoning, Luo et al. synthesized PMAO (poly(maleic anhydride-alt-1-octadecene))–PEG-coated ultra-small IONPs, onto which OVA was conjugated covalently, assessing their efficacy as a prophylactic and therapeutic vaccine for malignant melanoma. When used as therapy, subcutaneous injection of IONPs alone in tumor-bearing mice delayed both primary OVA-expressing B16F10 tumor growth and the number of lung metastases; when conjugated with OVA, however, a significantly greater inhibition was observed [179]. Interestingly, while prophylactic injection of OVA alone delayed the appearance of OVA-expressing B16F10 tumors, the use of OVA-PMAO-PEG@IONPs completely inhibited primary tumor growth and the onset of metastatic lung nodules. Therefore, the influence that the variable intrinsic biological activities of IONPs have on macrophage-activation status makes IONPs instrumental for developing combinatorial immunotherapy approaches. 

#### 4.1.3. IONPs and Dendritic Cells (DC)

Dendritic cells (DCs) are another important cellular target for IONP-based immunomodulatory therapies. They are the primary antigen-presenting cells in the organism and represent the link between the innate immune system, which acts as the first line of defense by detecting external threats, and the adaptive immune system, which responds to the pathogen by mounting immune memory responses of exquisite specificity [180]. In their immature state, DCs scan the microenvironment for danger using pathogen recognition receptors that bind pathogen-associated molecular patterns (PAMPs) [181]. Once an immature DC recognizes a PAMP, it becomes activated and matures into a professional antigen-presenting cell that is capable, among other things, of priming naïve T cells. DCs are therefore critical for mounting potent and durable immune responses to pathogens. DC theragnosis with IONPs thus represent an attractive approach for immunomodulation of antitumor immune responses, although strategies need to take into consideration the activation status of the DC. Indeed, Mou et al. found that while labeling mature DCs with IONPs does not have a significant effect on mature DC behavior, IONP-loaded immature DCs became activated as measured by increased CD80, CD86, and MHC-II expression. IONPs may influence the antigen-presentation function of DCs. On this issue, Shen et al. observed that lactosylated N-alkyl-polyethyleneimine (PEI_2k_)-IONPs promoted DC maturation through a mechanism involving NP-mediated induction of protective autophagy [182]. Likewise, Liu et al. demonstrated that increasing concentrations of pristine IONPs enhanced OVA cross-presentation in a model of DC. Curiously, the positively charged aminopropyltrimethoxysilane (APTS)-coated IONPs appeared to promote more efficient antigen cross-presentation as compared to the negatively charged IONPs (DMSA-coated IONPs), and this was dependent on TLR-3 [183]. This adjuvant effect of IONPs was also demonstrated by Zhao et al. in an OVA-based vaccine model by administering OVA@IONPs to OVA-expressing CT26 tumor-bearing mice, which produced a significant delay in tumor growth [184]. Zhang et al., however, revealed that PEG-coated IONPs disturbed mitochondrial dynamics through an increase in autophagy, and as a consequence, treated immature DCs exhibited downregulation of co-stimulatory molecules such as CD86, CD80, and CCR7, as well as reduced phagocytic capacity [185]. Therefore, as seen in macrophages, the effects of IONPs on DCs is variable and depends on a plethora of factors such as IONP size, shape, and coating, as well as DC maturation status, among others. Modulation of DC activity through IONP treatment is therefore a promising area of research that will require continued efforts to pinpoint the critical factors influencing IONP–DC interactions.

### 4.2. Iron Oxide and Functions of Endothelial Cells

Although myeloid cell interaction with IONPs is essential to understand, design, and improve IONP-based theranostics, endothelial cells (ECs) also impact the efficacy of such approaches, as these cells necessarily interact with IONPs when migrating to the interstitial and local microenvironment. As major targets of oxidative stress, ECs can engage anti-oxidant mechanisms that protect them from apoptosis. Thus, even in the presence of IONPs acting as ROS-triggering agents, ECs can promote anti-oxidant protective mechanisms. Duan et al. demonstrated that dextran-coated IONPs induced autophagy in human umbilical vascular endothelial cells (HUVECs), which in turn promoted cell survival. These IONP-treated HUVECs exhibited resistance to H_2_O_2_-induced cell death [186]. Likewise, Zhang et al. found that pristine IONPs disturbed autophagy in HUVECs and exacerbated the production of pro-inflammatory cytokines such as IL-1β and TNFα [187].

We also showed that polyethyleneimine (PEI)-coated IONPs profoundly alter EC function, which indicated that IONP-based reagents could be designed to modulate angiogenesis. PEI-coated IONPs disturbed the formation of focal adhesions and inhibited cell migration and in vitro tube formation through ROS-associated responses. Consistent with these in vitro effects, in vivo administration of PEI-coated IONPs reduced the number of vessels in a human breast cancer model [119]. ROS also mediates polyglucose sorbitol carboxymethyether (PSC)-coated IONP-triggered induction of epithelial-to-mesenchymal transition (EMT) in vascular ECs. Wen et al. observed that PSC-coated IONPs reduced the formation of tubules in vitro, closely resembling what we observed with PEI-coated IONPs; in contrast to our data, however, the authors observed enhanced EC migration [188]. It is therefore likely that the synthetic identity of IONPs also influences the EC response to these nanoreagents. Investigating the clinically relevant contrast agent, Endorem^®^ (dextran-coated IONPs), and custom-made silica@IONPs, Atanina et al. observed that treatment with these nanosystems decreased impedance, and thus integrity, of human microvascular endothelial-cell layers without affecting their viability. The loss of EC layer integrity was accompanied by the appearance of surface intercellular gaps and a decrease in NO production [189]. Altogether, it appears that IONPs mostly impair EC functions, suggesting they could potentially be used as an anti-angiogenic factor. However, just like macrophages, the EC response to IONPs depends greatly on several factors, including synthetic identity. Matuszak et al. proved that lauric acid-coated and BSA-stabilized IONPs are highly internalized by ECs, leading to acute toxicity, while lauric acid/BSA-coated and dextran-coated IONPs exhibited no evident toxicity [190]. The effects of IONPs on EC remains a somewhat underexplored area of knowledge. Given the importance of these cells in modulating immune responses and their presence at the interface between IONPs and the tumor environment, further insight into the intrinsic activity of IONPs on ECs will be of great interest to improve theranostic applications.

### 4.3. Tumor Microenvironment and Iron Oxide Nanoparticles 

At this point, we have only discussed the direct implications that IONP loading has on cells of the mononuclear phagocytic system, dendritic cells, and endothelial cells. Nonetheless, when analyzing the tumor microenvironment (TME), we should take into consideration its intrinsic complexity. As a mere reminder, TME is directly linked to a plethora of biological mechanisms that support tumor initiation, progression, and metastasis [191,192]. Processes such as proliferative [193,194], anti-apoptotic [195], pro-angiogenic [196], and immune-suppressive [197] phenomena, as well as mechanisms related to immune-surveillance evasion by tumors [198] greatly depend on the composition and organization of the TME. The niche that comprises the TME is formed by immune and endothelial cells as well as fibroblasts, and all have the potential to interact with IONPs that reach the tumor mass. Therefore, IONPs are expected to exert a biological impact on these cells as well as the tumor cells, which are usually the main targets of IONP-based theranostics. We have demonstrated that PEI-coated IONPs disturbed invadosome formation by the mouse tumor cells Pan02, and, as a consequence, inhibited tumor cell migration and invasion [122]. Moreover, these same PEI-coated IONPs altered macrophage and endothelial-cell activity in vitro and in vivo [119,122], illustrating the feasibility of developing nanoreagents that impair tumor cell biology, modify immune infiltration, and alter tumor angiogenesis. 

The presence of iron ions can also modulate the activity of the TME. Costa da Silva et al. showed that the presence of iron-loaded macrophages nesting in the invasive margins of non-small lung cell tumors correlated with smaller tumor size [199]. These iron-loaded cells localized near the sites of red blood cell (RBC) extravasation, thus pointing to RBCs as the iron source. More precisely, hemolytic RBCs trigger a TAM polarization toward an M1-like phenotype as measured by mRNA expression of M1 markers (*Il6*, *Nos2*, and *Tnfa*), and increased anti-tumor activity [199]. Costa da Silva et al. also found that cross-linked iron oxide nanoparticles injected intravenously in Lewis lung carcinoma (LLC)-bearing mice accumulated within F4/80 macrophages and reduced tumor growth [199]. These findings are of substantial consequence for IONP-based cancer theragnosis [200] as they indicate that, should IONPs accumulate within TAM in the tumor margins of inner zones, TAMs could revert their phenotype from M2 to M1.

IONPs can also support the anti-tumor effect by enhancing antigen cross-presentation in the tumor niche, as demonstrated by Lee et al. [201]. This enhancement was attributed to a mere increase in antigen delivery to DCs as compared to antigen alone, and not to the intrinsic biological effects of the carriers, SiO_2_@IONPs, on DC activation status. Thus, the adjuvancy of IONPs was more physical than biological, i.e., facilitating antigen endocytosis [201]. Another study using more complex nanocomposites in which an OVA antigen was covalently attached to IONPs showed a drastic reduction of OVA-expressing B16 tumor-derived lung metastasis in vivo [179]. In this work, IONPs exhibited an anti-tumor effect when injected alone, suggesting they also possess intrinsic biological activity that mitigates tumor growth. Similarly, Zanganeh et al. showed that the FDA-approved IONP, ferumoxytol, displayed in vivo anti-tumor effects in a mouse breast cancer model, effects which were most likely mediated through the induction of pro-inflammatory macrophages in the TME [176]. IONPs can also directly alter tumor biology. The therapeutic value of IONPs, therefore, should not rely solely on the capacity of these nanoparticles to modulate the tumor cell biology, but rather should also take into account their effects on the TME. 

## 5. Conclusions

IONPs have shown great potential as theranostic agents for cancer treatment and imaging. This is due in part to their biocompatibility on account of the existence of iron metabolism in the organism, which can eliminate the putative excess iron that results from the degradation of these nanomaterials. As part of the antitumor focus taken in the present review, we have seen that IONPs will primarily encounter cells of the mononuclear phagocytic system, dendritic cells, endothelial cells, and tumor cells. The physical, synthetic (coating), and biological identity of IONPs will influence their effects on the biology of the cells they encounter in the organism; conversely, the cell type and programming encountered by these nanoparticles will influence the effects triggered by IONPs. 

The degradation of the NP iron core can influence cellular iron metabolism, which in turn can affect activation status. Synthetic identity, including surface modifications, is also critical for determining IONP interaction with cell types; by way of example, the synthetic identity of IONPs can be modified to alter the internalization mechanism of cells. Overall, some common features emerge when assessing the cellular effects of IONPs. IONPs can (1) alter iron metabolism, (2) promote ROS production, and (3) likely induce autophagic machinery upon internalization. These complex interactions between the nanomaterial identities and the extra- and intra-cellular environment will ultimately define the intrinsic biological identity of IONPs. Understanding these interactions will, in turn, allow for the development of more rational combinatorial nanoreagents for theranostics.

## Figures and Tables

**Table 1:** Nanoparticle identities and their outcomes.

Identity	Concept
**Physical**	This refers to the basic physical properties that define the nanoparticle core, e.g., superparamagnetism, plasmonic, or fluorescence [23,24].
**Synthetic**	Refers to the intrinsic physicochemical properties of the engineered surface coating, as well as its size, shape, and surface chemistry post-synthesis (surface coating modifications) [25,26,27,28].
**Biological**	Refers to the size and aggregation state of the nanoparticles in physiological fluids (i.e., blood, tissue micro-environment, intracellular space) and the biomolecule (e.g., protein) corona. Biological identity varies with changes in synthetic identity, microenvironment, and interaction time [27,28,29,30].

**Table 2:** List of some of the IONPs investigated for their protein corona composition. Only significantly enriched proteins are listed.

Iron Oxide Nanoparticle	Physical Identity	Synthetic Identity (Surface Coating)	Biological Fluid	Biological Identity
IONP@Glu [100]	Fe_3_O_4_	Poly(maleic anhydride-alt-1-octadecene)-EDC-glucose	Serum protein	Protein AMBP Coagulation factor XI Fibrinogen beta chain C4b-binding protein α-like Profilin-1
IONP@PEG [100]	Fe_3_O_4_	Poly(maleic anhydride-alt-1-octadecene)-EDC-PEG	Serum protein	Actin, aortic smooth muscle Keratin, type I cytoskeletal 10 Keratin, type II cytoskeletal 7 Lysozyme C Fructose-biphosphate aldolase
IONP@PMAO [100]	Fe_3_O_4_	Poly(maleic anhydride-alt-1-octadecene)	Serum protein	Fibrinogen α-chain Tubulin α-4A chain Adenylyl cyclase-associated protein Macrophage migratory inhibitory factor Ectonucleotide Pyrophosphatase/phosphodiesterase family Member 2
ZW-L1@PAA-USPIONs [104]	Fe_3_O_4_	ZW-L1@PAA	80% human serum	α-2-Macroglobulin precursor Apolipoprotein C-II precursor
ZW-L2@PAA-USPIONs [104]	Fe_3_O_4_	ZW-L2@PAA	80% human serum	Apolipoprotein C-II precursor Apolipoprotein A-I preprotein Apolipoprotein A-II preprotein Apolipoprotein A-IV precursor Serum albumin preprotein
ZW-L3@PAA-USPIONs [104]	Fe_3_O_4_	ZW-L3@PAA	80% human serum	Apolipoprotein B-100 precursor Vitronectin precursor Complement C3 precursor Serum albumin preprotein Apolipoprotein A-II preprotein
PAA@USPIONs [104]	Fe_3_O_4_	PAA	80% human serum	α-2-Macroglobulin precursor Apolipoprotein A-I preprotein Apolipoprotein C-III precursor Complement C3 precursor Apolipoprotein B-100 precursor
Rh-Citrate@ IONPs [105]	Fe_3_O_4_	Rhodium citrate	Human blood serum	Human serum albumin Complement C5 A-Kinase anchor protein 13 Apolipoprotein A-I α-2-HS-glycoprotein
@IONPs/PVP @IONPs/PEG @IONPs [95]	Fe_3_O_4_	Polyvinylpyrrolidone (PVA) or polyethylene glycol (PEG)	Human plasma	14-3-3-Protein β/α 14-3-3-Protein ε Protein kinase C inhibitor protein 1 78 kDa glucose-regulated protein (GRP-78) Actin, aortic smooth muscle (α-actin-2)
CSNP [97]	Fe_3_O_4_	Silica	Human plasma	Fibrinogen b Fibrinogen g Fibrinogen a Vitronectin Histidine-rich glycoprotein
Nanomag-D@SPIO [97]	Fe_3_O_4_	Dextran	Human plasma	Kininogen 1 microtubule-associated ser/thr Kinase-like Actin, beta Integrin, alpha 2b Pro-platelet basic protein

**Table 3:** Examples of IONPs and the endocytic pathways that mediate IONP internalization. IONPs are defined according to their identities as referred to in Table 1. The proposed receptors mediating their endocytosis are listed. (N.A.: not available).

IONP Name	Physical Identity	Synthetic Identity (Surface Coating)	Biological Identity	Endocytic Pathways	Receptors	Cell Type	References
Silica@IONP	Fe_2_O_3_	SiO_2_	N.A.	Caveolin-dependent	CDC42	HeLa	[120]
PEI@SPIONs	Fe_3_O_4_	Polyethyleneimine (PEI)	N.A.	Clathrin-dependent and caveolin-dependent	TLR4	RAW264.7 and Pan02	[121,122]
FA–PEI@SPIONs	Fe_3_O_4_	PEI	Folic acid	Clathrin-dependent	Folic acid receptor	HeLa	[123]
BP-D@IONPs	Fe_2_O_3_	DMSA and BODIPY	With/out 10% serum (aggregates)	Endocytosis-independent and clathrin-dependent	N.A.	Oligodendroglial (OLN-93)	[124]
Ferumoxides	Fe_3_O_4_	Dextran	N.A.	Clathrin-dependent	SR-A	THP-1	[125]
DMSA@SPIONs	Fe_3_O_4_	DMSA	N.A.	Clathrin-dependent (<200 nm) and macropinocytosis (aggregates > 200 nm)	N.A.	MCF-7	[126]
Carboxydextran@USPION	Fe_3_O_4_	Carboxydextran	N.A.	Clathrin-dependent	SR-A	Human macrophages	[127]
PLL@IONPs	N.A.	Poly-L-lysine	N.A.	Clathrin-dependent	TfR	HeLa	[128]
Carboxymethyl-dextran@IONPs	N.A	Carboxymethyl-dextran	Serum (protein corona)	Clathrin-dependent and caveolin-dependent	N.A.	CaCo-2	[129]
Aminosilane@IONPs	Fe_3_O_4_	Aminosilane	N.A.	Phagocytosis	N.A.	Lung cancer cell, SPC-A1	[130]
DMSA@IONPs	γ-Fe_2_O_3_	DMSA	N.A.	Clathrin-dependent, caveolin-dependent, and macropinocytosis	N.A.	RAW264.7	[131]
Maghemite–rhodium citrate NPs	γ-Fe_2_O_3_	Rh-citrate	N.A.	Clathrin-dependent	N.A.	MCF-7, MDA-MB-231, and HNTMCs	[132]
Aminodextran@IONPs	Fe_3_O_4_	Aminodextran	N.A.	Macropinocytosis	N.A.	A-549	[133]
PEI@IONPs	Fe_3_O_4_	PEI	N.A.	Adsorptive endocytosis	N.A.	RAW264.7	[116]
PEG@IONPs	Fe_3_O_4_	Polyethylene glycol	N.A.	Receptor-mediated endocytosis	N.A.	RAW264.7	[116]

**Table 4:** A list of IONPs recently investigated for their effect on the macrophage.

Iron Oxide Nanoparticles	Physical Identity	Synthetic Identity (Surface Coating)	Cell Type	Described Effects	Mechanism
Carboxymaltose@Fe_2_O_3_ [153]	Fe_2_O_3_	Carboxymaltose	J774A.1 Primary macrophages	Inhibits LPS-induced NO Inhibits IL-6 and TNFα secretion Hampers phagocytosis	Decreased free glutathione
PMA@IONPs (4 and 14 nm) [154]	Fe_3_O_4_	PMA		Hamper cell viability Promote extensive vacuolization Induce TNFα, CD86 and inhibit CD206 gene expression	Promotion of extensive vacuolization
PEGylated PMA@IONPs (4 and 14 nm) [154]	Fe_3_O_4_	PEGylates PMA	RAW264.7	Promote cell proliferation Promote extensive vacuolization Induce TNFα, CD86 and inhibit CD206 gene expression	Promotion of extensive vacuolization
PDSCE@IONPs [155]	γ-Fe_2_O_3_	Polydextrose sorbitol carboxymethyl-ether	In vivo and RAW264.7	Reduce the level of LPS-induced injury Induce a large amount of IL-10 Trigger autophagy	Promotion of autophagy through Cav1-Notch1/HES1
Carboxydextran@IONPs [156]	Fe_3_O_4_	Carboxydextran	In vivo local administration and J774.2	Downmodulate CD86, MHC-II, Arg1 and CD163 expression (transient) Hamper phagocytosis (transient)	N.A.
SiO_2_@IONPs [157]	γ-Fe_2_O_3_	SiO_2_	Peritoneal macrophages	Increase γH_2_AX (marker for double-strand break) Increase IL-10 production	N.A.
Resovist [158]	Fe_3_O_4_ γ-Fe_2_O_3_	Carboxydextran	Primary macrophages and RAW264.7	Induce autophagy Induce pro-inflammatory gene expression (TNFα, IL-12, MIP-1-α, etc.)	Promote autophagy through TLR4-p38-Nrf2-p62 signaling pathway
Feraheme [158]	Fe_3_O_4_	Carboxymethyl dextran		Induce autophagy Induce pro-inflammatory gene expression (TNFα, IL-12, MIP-1-α, etc.)	Promote autophagy through TLR4-p38-Nrf2-p62 signaling pathway
DMSA@IONPs [159]	Fe_3_O_4_	DMSA	RAW264.7	Induce pro-inflammatory cytokines Promote cell proliferation Promote macrophage migration Promote macrophage-driven Hepa1-6 cell killing	N.A.
PEI@IONPs [121]	Fe_3_O_4_ γ-Fe_2_O_3_	PEI	RAW264.7, THP-1, and primary peritoneal macrophages	Induce pro-inflammatory cytokines (IL-12, IL-1β, TNFα, etc.) Activate macrophages (increase CD40, CD80, CD86 and I-A/I-E) Activate the MAPK-dependent pathway Promote podosome formation and reduce ECM degradation	At least part of the effects are mediated by production of ROS and activation of TLR-4
Citrate@Fe_3_O_4_ of different shape (octopod, plate, cube, sphere) [160]	Mn-doped Fe_3_O_4_	Citrate	Bone marrow-derived macrophages (BMDMs)	Activate inflammasome (IL-1β) Induce pyroptosis Induce ROS production In this order: Octopod > plate > cube > sphere	Lysosome damage, ROS production, and K^+^ efflux, partially mediated by NLPR3
Alkyl-PEI@IONPs (30, 80, and 120 nm) [161]	Fe_3_O_4_	Alkyl-PEI	BMDMs	Induce IL-1β nm > 80 nm > 120 nm) Lysosome damage ROS production	Modulated by ROS
Fe_2_O_3_@D-SiO_2_ [162]	Fe_2_O_3_	SiO_2_	RAW264.7	Increase CD80, CD86 and CD64	Activate NF-κB and IRF5
Fe_3_O_4_@D-SiO_2_ [162]	Fe_3_O_4_	SiO_2_	RAW264.7	Negligible effect	N.A.
DMSA@IONPs [163]	Fe_3_O_4_	DMSA	M2-like THP1 BMDMs (M2)	Induce ROS production Change Fe metabolism to an iron-replete status Reduce Mac3, CD80 Increase IL-10 production Decrease migration but increase invasion	Activation of MAPK signaling
APS@IONPs [163]	Fe_3_O_4_	3-Aminopropyl triethoxysilane	M2-like THP1 BMDMs (M2)	Induce ROS production Change Fe metabolism to an iron-replete status Reduce Mac3, CD80 Increase IL-10 production Decrease migration but increase invasion	Activation of MAPK signaling
AD@IONPs [163]	Fe_3_O_4_	Aminodextran	M2-like THP1 BMDMs (M2)	Induce ROS production Change Fe metabolism to an iron-replete status Reduce Mac3 Decrease migration but increase invasion	Activation of MAPK signaling
